# Targeting of Mutant p53 and the Cellular Redox Balance by APR-246 as a Strategy for Efficient Cancer Therapy

**DOI:** 10.3389/fonc.2016.00021

**Published:** 2016-02-03

**Authors:** Vladimir J. N. Bykov, Qiang Zhang, Meiqiongzi Zhang, Sophia Ceder, Lars Abrahmsen, Klas G. Wiman

**Affiliations:** ^1^Department of Oncology-Pathology, Cancer Center Karolinska (CCK), Karolinska Institutet, Stockholm, Sweden; ^2^Aprea AB, Solna, Sweden

**Keywords:** APR-246, mutant p53, apoptosis, thioredoxin reductase, glutathione, redox balance, clinical trial, cancer therapy

## Abstract

TP53 is the most frequently mutated gene in cancer. The p53 protein activates transcription of genes that promote cell cycle arrest or apoptosis, or regulate cell metabolism, and other processes. Missense mutations in TP53 abolish specific DNA binding of p53 and allow evasion of apoptosis and accelerated tumor progression. Mutant p53 often accumulates at high levels in tumor cells. Pharmacological reactivation of mutant p53 has emerged as a promising strategy for improved cancer therapy. Small molecules that restore wild type activity of mutant p53 have been identified using various approaches. One of these molecules, APR-246, is a prodrug that is converted to the Michael acceptor methylene quinuclidinone (MQ) that binds covalently to cysteines in p53, leading to refolding and restoration of wild type p53 function. MQ also targets the cellular redox balance by inhibiting thioredoxin reductase (TrxR1) and depleting glutathione. This dual mechanism of action may account for the striking synergy between APR-246 and platinum compounds. APR-246 is the only mutant p53-targeting compound in clinical development. A phase I/IIa clinical trial in hematological malignancies and prostate cancer showed good safety profile and clinical effects in some patients. APR-246 is currently tested in a phase Ib/II trial in patients with high-grade serous ovarian cancer.

## Introduction

Recent DNA sequencing of 3281 human tumors within The Cancer Genome Atlas (TCGA) has confirmed the high frequency of TP53 mutations in cancer. At least 42% of the cases of 12 common human tumor types carry mutant TP53 (1). In high-grade serous (HGS) ovarian cancer, the fraction of tumors with mutant TP53 is almost 95%. No other gene is mutated at such high frequency in cancer. See also TP53 databases p53.iarc.fr and p53.free.fr. The second and third most frequently mutated genes are PIK3CA that encodes the p110 alpha catalytic subunit of PI3 kinase and PTEN, a lipid phosphatase that regulates Akt kinase activation, which are mutated in 17.8 and 9.7% of the cases of the 12 common tumor types, respectively (1).

Wild type p53 protein induces cell cycle arrest, senescence, and apoptosis in response to cellular stress by upregulating target genes such as p21, Bax, Puma, and Noxa (2). p53 can also regulate cell metabolism and redox status through target genes such as TIGAR and GLS2 (3–5). It remains unclear exactly how p53 mediates potent tumor suppression. *In vivo* studies in mice have shown that certain engineered p53 mutants that fail to transactivate pro-arrest and pro-apoptosis target genes can still prevent tumor development (6, 7). Similarly, mice lacking the p53 target genes p21 that mediates p53-dependent cell cycle arrest and Puma and Noxa that mediate p53-dependent apoptosis do not show increased tumor incidence (8). These findings argue that other p53 transcriptional targets, for instance those involved in regulation of metabolism, are critical for p53-mediated tumor suppression.

Oncogenic stress as a result of oncogene activation or loss of cell cycle control characterizes early stages of tumor evolution. This leads to aberrant DNA replication, which triggers a DNA damage response (DDR) involving activation of ATM, Chk1 and Chk2 kinases, and p53, and induction of senescence or apoptosis (9). Activation of DDR and p53 upon oncogenic stress serves to eliminate incipient tumor cells and forms a critical barrier against tumor development. DDR inactivation by mutation in ATM or TP53 allows cell survival and tumor progression. Many TP53 mutations are missense mutations resulting in amino acid substitutions in the DNA-binding core domain and disruption of p53-specific DNA binding and transcriptional transactivation (10). Loss of wild type p53 is associated with increased resistance to chemotherapy.

The high frequency of missense TP53 mutations in human tumors and the fact that mutant p53 often accumulates at high levels in tumor cells make mutant p53 a potential target for improved cancer therapy. Pharmacological reactivation of mutant p53 would restore p53-dependent senescence and apoptosis, and presumably also p53-mediated regulation of metabolism and other processes, and thus eliminate tumors *in vivo*. Indeed, studies in various mouse models have demonstrated that restoration of wild type p53 expression *in vivo* leads to rapid tumor elimination (11–13). This suggests that restoration of functional p53 can trigger tumor cell death and lead to tumor clearance even if a tumor carries multiple genetic alterations that drive tumor growth.

## Pharmacological Reactivation of Mutant p53

A growing number of small molecules that can reactivate mutant p53 have been identified over the past 15 years, using either chemical library screening or rational drug design. These include CP31398, PRIMA-1Met/APR-246, PK-083, PK-5174, SCH529074, and NSC319726 (ZMC1). We have previously reviewed this field (14). This review is focused on PRIMA-1 (APR-017) and the structural analog PRIMA-1Met, now named APR-246, both of which were identified in our laboratory. As will be discussed below, both compounds are prodrugs that form the active moiety MQ. We will also highlight the clinical development of APR-246.

We identified PRIMA-1 in a screen of a small structurally diversified chemical library from NCI (Diversity set) for compounds that could induce cell cycle arrest or cell death preferentially in cells expressing mutant p53 (15). Cell growth and viability were assessed by the WST1 assay. PRIMA-1 showed the strongest preference for mutant p53-expressing cells and was selected for further studies. Experiments with antibodies specific for correctly folded wild type p53 (PAb1620) or unfolded mutant p53 (PAb240) revealed that PRIMA-1 could induce refolding of mutant p53 and enhance mutant p53 DNA binding in gel shift assays. PRIMA-1 treatment of tumor cells carrying various mutant p53 resulted in upregulation of p53 target genes such as p21, Bax, and Mdm2, and induction of cell death by apoptosis. Systemic administration of PRIMA-1 in mice carrying Saos-2-His273 tumor xenografts demonstrated significant inhibition of xenograft tumor growth *in vivo* (15). In parallel, our analysis of available data in the NCI database confirmed that PRIMA-1 preferentially targets tumors cells carrying mutant p53 and has an activity profile that is entirely distinct from those of commonly used chemotherapeutic drugs such as cisplatin and 5-FU (16). Subsequently, the structural analog PRIMA-1Met or APR-246 that has superior permeability properties was identified. APR-246 was shown to synergize with chemotherapeutic drugs, e.g., adriamycin and cisplatin (17).

## Targeting Mutant p53 by Michael Addition

**Figure 1:**
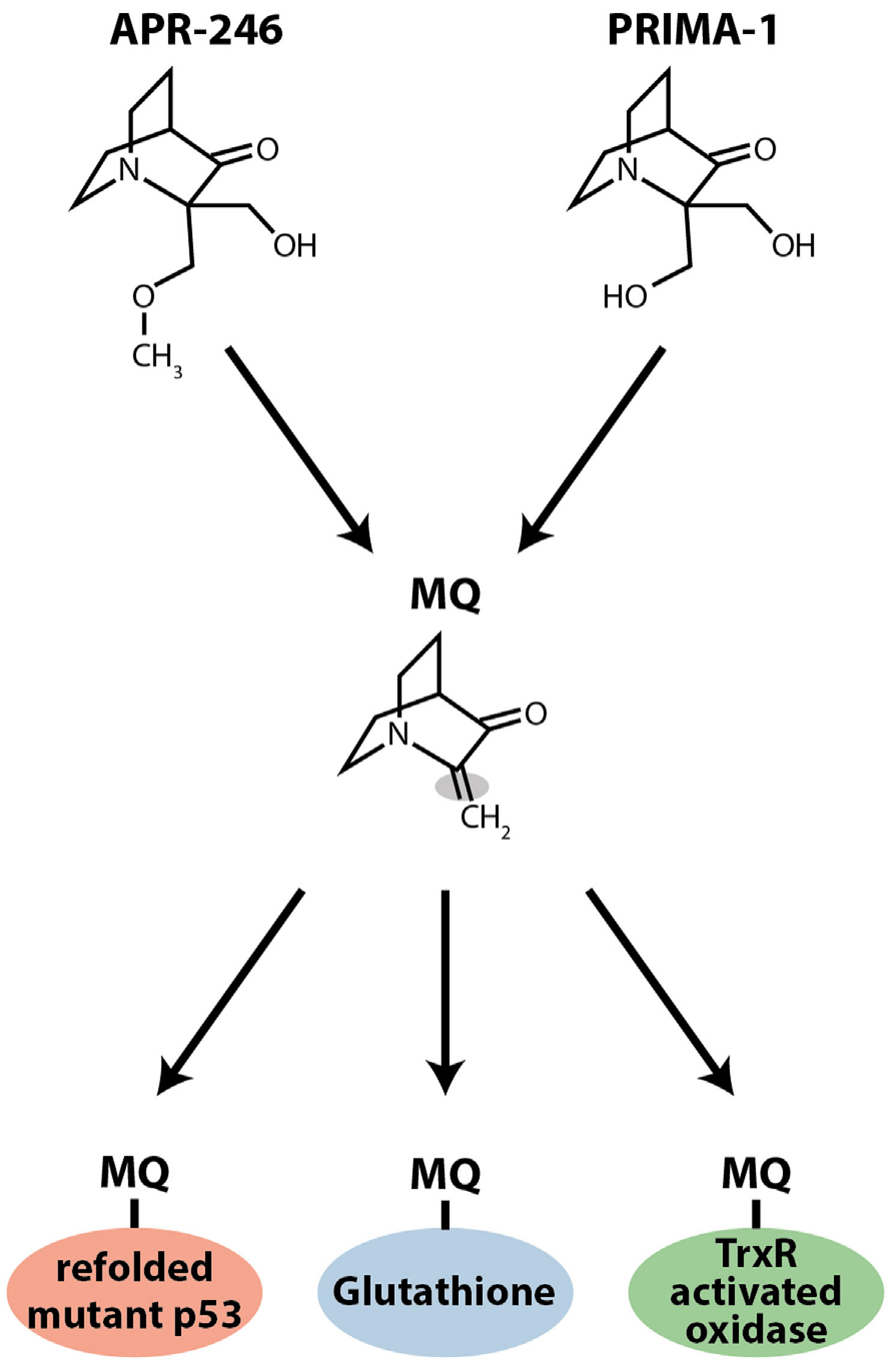
**Chemical structures of PRIMA-1 and APR-246 (PRIMA-1Met)**. Both compounds form the Michael acceptor methylene quinuclidinone (MQ), which is the active moiety. MQ binds covalently to thiols in mutant p53. MQ also targets thioredoxin reductase (TrxR) and glutathione (GSH). MQ binding to TrxR converts the enzyme to an active oxidase, which generates ROS, and MQ binding to glutathione depletes intracellular free glutathione, which also induces ROS.

Our data clearly showed that PRIMA-1 and APR-246 were able to reactivate various forms of mutant p53 and trigger tumor cell apoptosis, but their molecular mechanism of action remained obscure. However, we found that both compounds are converted to methylene quinuclidinone, MQ, a Michael acceptor that can react with soft nucleophiles such as thiols in proteins (Figure 1). The p53 core domain has 10 cysteine residues. Mass spectrometry demonstrated that MQ binds covalently to the p53 core domain (18). Several findings support the notion that MQ binding to p53 is critical for the effect of PRIMA-1 and APR-246. *N*-acetylcysteine (NAC), a thiol group donor, blocks PRIMA-1-induced apoptosis and PRIMA-D (APR-320), a structural analog that cannot be converted to MQ, has no effect on tumor cells at concentrations corresponding to those used for PRIMA-1 and APR-246. Moreover, transfer of MQ-modified mutant p53 protein into p53 null tumor cells induces expression of p53 target genes and cell death by apoptosis (18). These results demonstrate that MQ is the active compound and that MQ-modification of mutant p53 *per se* is sufficient to induce tumor cell death. Thus, PRIMA-1 and APR-246 are prodrugs that form the biologically active compound MQ (Figure 1). This conversion is spontaneous and occurs over a time frame of a few hours at physiological pH (18). Since MQ is reactive, its administration as a prodrug is probably critical in order to avoid adduct formation with various extracellular targets.

It is interesting to note that MIRA-1, another compound identified in our screen of the NCI Diversity set, is a maleimide with known Michael acceptor activity. Moreover, Kaar and Fersht and their colleagues identified a series of Michael acceptors that bind covalently to both wild type and mutant p53 core domains, resulting in increased protein melting temperature. Analysis of the reactivity of the cysteines in p53 by mass spectrometry revealed preferential reaction with C124 and C141, followed by C135, C182, and C277, and then C176 and C275 (19). These results further support the idea that adduct formation at cysteines can stabilize the native conformation of p53.

Among the 10 cysteine residues in p53’s core domain, four – Cys182, Cys229, Cys242, and Cys277 – are exposed on the surface of the protein and accessible for modification in correctly folded p53 (20, 21). Presumably, additional cysteines are exposed in unfolded mutant (or wild type) p53, allowing more extensive thiol modification (18). Computational analysis of structural p53 models identified a binding pocket between the L1 loop and S3 sheet in the p53 core domain, containing cysteines C124, C135, and C141 (22). Docking analysis indicated that MQ, as well as other thiol-targeting compounds including MIRA-1, can bind to the L1/S3 pocket. These results were validated in living cells by introduction of a C124A substitution in R175H mutant p53. Indeed, C124A substitution abolished the apoptotic effect of PRIMA-1 in Saos-2 osteosarcoma cells expressing R175H mutant p53.

Thus, APR-246/MQ, MIRA-1 and the compounds identified by Kaar et al. (19) share a common chemical property and presumably promote refolding of mutant p53 by a similar mechanism. The ability to modify cysteines in mutant p53 distinguishes APR-246/MQ from compounds like PK-083 and PK-7088 that bind to a crevice in the Y220C mutant p53 protein and raise its melting temperature (23). APR-246 also has a different mechanism of action than the compound NSC319726 (ZMC1), a zinc chelator that refolds His175 mutant p53 as well as several other mutant p53 proteins (24). Clearly, mutant p53 refolding and reactivation can be achieved by various molecular strategies. Some strategies work for specific mutant forms of p53, whereas other strategies are applicable to a range of mutant p53 proteins.

## APR-246 Reactivates Mutant Forms of p53 Family Members p63 and p73

p53 is a member of a protein family with two other members, p63 and p73 (25). In contrast to TP53, neither the TP63 nor TP73 genes are mutated at any significant frequency in human tumors. However, TP63 missense mutations occur in certain developmental syndromes such as the Ectrodactyly–ectodermal dysplasia–cleft (EEC) syndrome (26). All three proteins share a high degree of sequence similarity in the DNA-binding core domain (25). The 10 cysteines in the p53 core domain are all conserved in both p63 and p73. This raises the question as to whether APR-246 can affect mutant p63 and/or p73 folding and activity. We first examined the effect of APR-246 on human tumor cells carrying exogenous temperature-sensitive missense mutant TP63 and TP73. APR-246 induced the expression of p53/p63/p73 target genes, cell cycle arrest, and cell death by apoptosis in these cells (27). To assess the effect of APR-246 on mutant p63 in a more physiological context, we used human keratinocytes derived from EEC patients carrying R204W or R304W mutant TP63. These two TP63 mutants correspond to the tumor-associated hot spot TP53 R175H and R273H mutants. Treatment with APR-246 led to increased expression of p63 target genes and at least a partial rescue of keratinocyte differentiation (28). Similarly, APR-246 rescued corneal differentiation in iPS cells from EEC individuals (29). Thus, the targeting of mutant versions of the two structurally related transcription factors p63 and p73 by APR-246 leads to entirely different biological responses that recapitulate the normal functions of each protein. These results argue convincingly that the biological effects of APR-246 are mediated by direct binding to mutant p53 or p63 and refolding the mutant proteins into an active conformation.

## APR-246/MQ Targets Components of the Cellular Redox System

The observation that MQ can bind to thiols suggested that it might also target thiol-containing redox regulators such as glutathione and thioredoxin. Indeed, we found that APR-246 is a potent inhibitor of thioredoxin reductase (TrxR1), a selenocysteine-containing enzyme that catalyzes the reduction of thioredoxin (30). APR-246 inhibits the activity of TrxR1 both *in vitro* and in living cells. This effect is presumably mediated through modification of the selenocysteine residue in TrxR1 by MQ. MQ binding converts TrxR1 into an NADPH oxidase that contributes to ROS production and cell death induced by APR-246 (30).

Methylene quinuclidinone has also been shown to bind to cysteines in glutathione (GSH), leading to a decrease in free intracellular glutathione concentrations and increased ROS levels (31, 32). Since glutathione can mediate resistance to platinum drugs by conjugation and export, this effect of MQ may at least in part account for the strong synergy between APR-246 and platinum drugs (see below). APR-246 did not inhibit GCLM (regulatory subunit of γ-glutamyl cysteine-synthase) or GSS (glutathione synthetase) in the GSH synthesis pathway, indicating that the observed GSH depletion is not caused by decreased synthesis (32).

**Figure 2:**
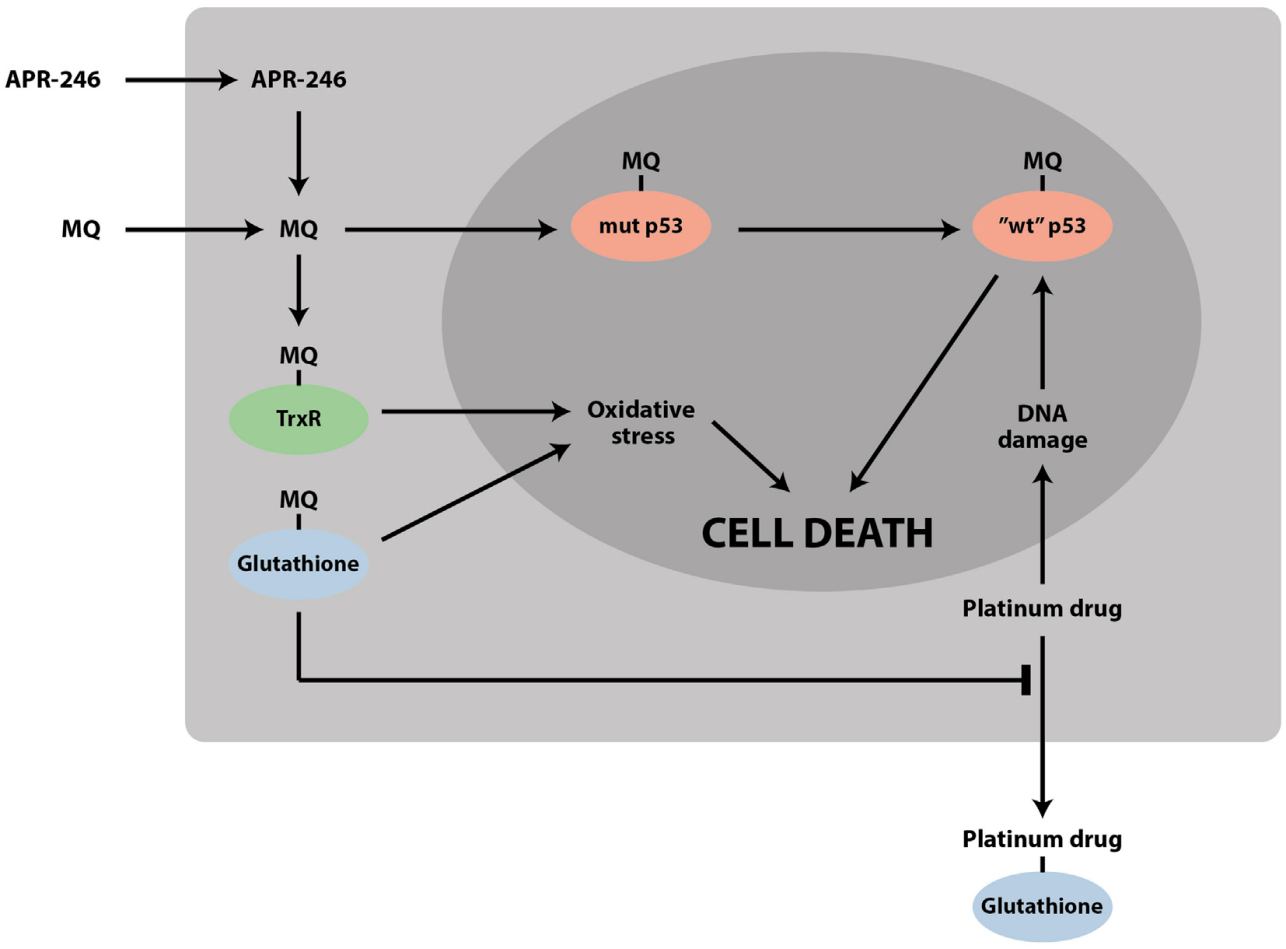
**APR-246/MQ targets both mutant p53 and the cellular redox balance**. The striking synergy between APR-246 and DNA-damaging chemotherapeutic drugs such as cisplatin and doxorubicin can be due to (1) refolding and reactivation of mutant p53 by APR-246, which enhances the response to DNA-damaging agents; (2) accumulation of mutant p53 by DNA-damaging agents, which potentiates the effect of APR-246; (3) depletion of glutathione by MQ, which inhibits efflux of drugs such as cisplatin; and (4) inhibition of TrxR and conversion of the enzyme to an active oxidase, leading to increased oxidative stress.

Thus, accumulating data on the effects of PRIMA-1/APR-246 on the cellular redox balance demonstrate that these compounds have a dual mechanism of action that targets two Achille’s heels of tumor cells: mutant p53 and the redox balance (Figure 2). The targeting of these two pathways may allow more efficient elimination of tumor cells and lower the probability of resistance development. This dual mechanism provides an explanation for reported mutant p53-independent effects of APR-246.

## Effects on Mutant and Wild Type p53

Methylene quinuclidinone can bind to both wild type and mutant p53 (18), and it is conceivable that MQ binding can induce refolding of misfolded wild type p53 in tumor cells. However, available data so far indicate that APR-246/MQ has little toxicity in normal cells. Wild type p53 is expressed at low levels in most normal cells and tissues in the absence of stress, whereas many tumor cells express high levels of unfolded mutant p53. Also, normal cells have a higher capacity to cope with oxidative stress as compared to tumor cells (33). While MQ binding to mutant p53 can restore p53-dependent apoptosis (18), MQ binding to other cellular proteins may not necessarily have major effects on cell growth and survival, except for binding to TrxR and GSH and possibly other anti-oxidative proteins, as discussed above. The benign safety profile of APR-246 observed in the first clinical study (34) is consistent with the lack of major toxicity in normal cells.

Interestingly, the response of wild type and mutant TP53-carrying tumor cells to MQ is enhanced by hypoxia (35). Hypoxia (≤1% oxygen) increased the sensitivity of SKBR3 cells (R175H mutant TP53) to PRIMA-1 treatment. In MCF-7 cells (wild type TP53), chemical hypoxia induced by CoCl_2_ led to accumulation of unfolded wild type p53, as assessed with the monoclonal antibody PAb240, and enhanced sensitivity to PRIMA-1. Presumably, this is due to MQ binding and refolding of unfolded “mutant-like” wild type p53 into an active conformation. The finding that hypoxia can potentiate the efficacy of PRIMA-1 has important clinical implications. Due to insufficient blood supply, rapidly growing tumors *in vivo* are often hypoxic, and it is conceivable that this could enhance the therapeutic efficacy of APR-246, both in wild type and mutant TP53-carrying tumors.

## Synergy with Conventional Chemotherapeutic Drugs and Novel Experimental Drugs

A major hurdle for achieving efficient elimination of tumors and long-term cancer cure is the rapid development of therapy resistance. There are numerous mechanisms for such resistance, including enhanced DNA repair and increased efflux of chemotherapeutic drugs from the tumor cell (36). The problem of resistance is relevant not only for conventional chemotherapeutic drugs but also for targeted drugs, as exemplified by resistance development in CML upon treatment with the novel drug imatinib (Gleevec) that inhibits the BCR-ABL kinase (37). Therefore, it is important to explore possible synergies between APR-246 and conventional anticancer agents, novel targeted drugs, and experimental drugs.

The DNA damage caused by chemotherapeutic drugs such as cisplatin and doxorubicin induces tumor cell death to a large extent via wild type p53 activation and p53-induced apoptosis. Accordingly, tumor cells carrying mutant p53 or completely lacking p53 are often more resistant to conventional chemotherapy. This suggests that restoration of wild type p53 function by APR-246 might synergize with, for example, cisplatin. Indeed, we and others have demonstrated strong synergy between APR-246 and chemotherapeutic drugs such as cisplatin, 5-fluorouracil (5-FU), and doxorubicin in mutant p53-carrying lung, ovarian, and esophageal cancer cells (17, 31, 38, 39). Synergistic effects have also been observed *in vivo* upon systemic administration (17, 39).

There are several possible reasons for the observed synergy (Figure 2). First, as alluded to above, restoration of wild type function to mutant p53 by APR-246 might increase sensitivity to chemotherapeutic drugs that depend on wild type p53 for induction of efficient tumor cell apoptosis. Second, treatment with cisplatin, adriamycin, or 5-FU leads to accumulation of mutant p53 (17, 39), which is expected to enhance the effect of APR-246. Third, we and others found that APR-246, via MQ, depletes intracellular GSH levels (31, 32). Since formation of adducts with GSH and extracellular export is one mechanism of cisplatin resistance, MQ-mediated GSH depletion is likely to sensitize tumor cells to cisplatin. Fourth, MQ-mediated inhibition of TrxR and conversion of the enzyme to an active oxidase (30) should induce ROS levels, which will further enhance DNA damage and p53-dependent cell death. Inhibition of TrxR will also negatively affect the activity of ribonucleotide reductase, needed for providing deoxyribonucleotides for DNA replication and repair (40).

In contrast to the mutant p53-dependent synergy of APR-246 with cisplatin and 5-FU, the synergy between APP-246 and epirubicin was p53-independent in esophageal cancer cells (39). This could be due to the redox effects of APR-246, including inhibition of TrxR and/or GSH depletion. Cisplatin and 5-FU, but not epirubicin, induced the expression of mutant p53 (39). Synergy has also been observed between APR-246 and the experimental compound RITA in AML cells. This synergy could arise from increased levels of mutant p53 upon induction of DNA damage by RITA (41).

In addition, APR-246 synergized with daunorubicin in AML cells carrying wild type p53 (41). Synergy in the absence of mutant p53 may be due to APR-246-mediated redox effects. However, as discussed above, MQ can also bind to wild type p53 and restore an active conformation under hypoxic conditions. There is evidence suggesting that wild type p53 may occur in a misfolded conformation in some tumors, e.g., B-CLL (42) and AML (43). This raises the possibility that refolding of wild type p53 by APR-246 may be responsible for synergy with chemotherapeutic agents in AML cells.

PRIMA-1 at 50 μM induced G2/M phase accumulation of parental mouse L1210 leukemia cells carrying mutant p53 but had only a minor effect on the cell cycle distribution of Y8 cells, a subline of L1210 that lacks p53. A striking synergistic induction of necrosis was observed in L1210 cells upon combination treatment with PRIMA-1 and the cyclin-dependent kinase inhibitor flavopiridol. However, in Y8 p53 null cells, combination of PRIMA-1 and flavopiridol caused a synergistic increase in apoptosis (44). Thus, combination treatment with PRIMA-1 (or presumably APR-246) can lead to cell death through alternative routes, depending on the presence or absence of mutant p53.

Mutant p53 reactivation by APR-246 leads to induction of the p53 target and antagonist Mdm2 (15), which promotes p53 degradation by the proteasome. Therefore, it is conceivable that inhibition of Mdm2-p53 binding and Mdm2-mediated p53 degradation might potentiate the effect of APR-246. Indeed, strong synergy was observed between PRIMA-1 and the Mdm2 inhibitor Nutlin-3 in pancreatic cancer cells (45). Moreover, gene therapy with the tumor suppressor gene FHIT (fragile histidine triad), whose gene product has been shown to inactivate Mdm2 (46), resulted in synergistic inhibition of tumor growth in combination with APR-246 (47). Since several compounds that disrupt p53-Mdm2 binding are now being tested in the clinic, these results may have profound implications for the future clinical use of both APR-246 and inhibitors of p53-Mdm2 binding.

## Clinical Development

APR-246 has been tested in a first-in-man phase I/IIa clinical trial in patients with hematological malignancies or hormone-refractory prostate cancer (34). The main aim was to determine maximum tolerated dose (MTD) of APR-246 and to assess safety and pharmacokinetic properties. Patients were not preselected based on TP53 mutation status. The treatment regimen was 2-h infusion of APR-246 for 4 days. Overall, the study showed that APR-246 is well tolerated and only relatively minor and transient side effects were observed, including dizziness, fatigue, headache, nausea, and confusion. MTD was defined as 60 mg/kg. Plasma concentrations of APR-246 reached 250 μM, well above concentrations required for robust induction of tumor cell apoptosis in cell culture experiments. Analysis of isolated patient leukemic cells by FACS revealed induction of p53 targets Bax and Puma upon APR-246 treatment, and microarray analysis showed substantial alterations in gene expression, including genes associated with cell cycle regulation and cell death, consistent with the proposed mechanism of action. Furthermore, one AML patient carrying V173M mutant TP53 showed a significant reduction in bone marrow blasts, and one patient with a TP53 splice site mutation had a minor response according to CT scan. Thus, APR-246 is safe and shows signs of clinical activity. APR-246 is currently being tested in combination with carboplatin and pegylated doxorubicin in a phase Ib/II clinical study in HGS ovarian cancer, a tumor type with a 95% frequency of TP53 mutations (see www.clinicaltrials.gov).

## Future Perspectives

The development of efficient mutant p53-reactivating anticancer drugs is expected to have a major impact on public health globally, given the high frequency of TP53 mutations in a wide range of human tumors. In certain tumor types, TP53 is mutated in the great majority of the cases. In general, clinical studies have shown that mutant TP53-carrying tumors respond less well to conventional chemotherapeutic drugs and have worse prognosis than wild type TP53-carrying tumors. The ongoing phase Ib/II clinical study with APR-246 will provide solid data on clinical efficacy in combination with standard chemotherapy. Importantly, the mechanism of action of APR-246 – i.e., dual targeting of both mutant p53 and the cellular redox system – suggests that APR-246 will synergize with many DNA-damaging chemotherapeutic drugs, and such synergy has been confirmed in a number of published studies. An important goal for further studies is to assess clinical efficacy in combination with relevant chemotherapeutic and targeted drugs in various tumor types. Ultimately, APR-246 may allow greatly improved therapy of a wide range of tumors that carry mutant TP53.

## Author Contributions

KW contributed to writing the manuscript and preparing the figures, communicated with the journal editor, and submitted the manuscript. VB, QZ, MZ, SC, and LA contributed to writing the manuscript and preparing the figures.

## Conflict of Interest Statement

Vladimir J. N. Bykov and Klas G. Wiman are co-founders and shareholders of Aprea AB, a company that develops p53-based cancer therapy, including APR-246. Klas G. Wiman is a member of its board. Lars Abrahmsen is employed by Aprea AB. The authors declare that the research was conducted in the absence of any commercial or financial relationships that could be construed as a potential conflict of interest.
